# Crystal structure and fluorescence properties of *catena*-poly[[(2,2′-bi-1*H*-imidazole-κ^2^ *N*,*N*′)cadmium]-di-μ-chlorido]

**DOI:** 10.1107/S2056989016013736

**Published:** 2016-09-09

**Authors:** Yang Liu, Hai-Hui Liu

**Affiliations:** a Key Laboratory of Functional Organometallic Materials, Department of Chemistry and Materials Science, Hengyang Normal University, Hengyang 421008, People’s Republic of China; b Department of Chemistry and Materials Science, Hengyang Normal University, Hengyang 421008, People’s Republic of China

**Keywords:** crystal structure, 2,2′-bi-1*H*-imidazole, cadmium, fluorescent quenching

## Abstract

In the polymeric title compound, [CdCl_2_(C_6_H_6_N_4_)]_*n*_, the central Cd^II^ atom is coordinated by four chloride ligands and two N atoms from a chelating 2,2′-bi-1*H*-imidazole mol­ecule, leading to a distorted octa­hedral Cl_4_N_2_ coordination set. As a result of the μ_2_-bridging character of the Cl ligands, chains parallel to the *c* axis are formed, with the chelating 2,2′-bi-1*H*-imidazole ligands decorated on both sides of the chain. The luminescence properties of the complex dispersed in di­methyl­formamide shows that the emission intensities are significantly quenched by nitro­benzene.

## Abstract 2

The title complex shows selective sensitivity to detecting nitro­benzene in DMF media due to the fluorescent quenching.

## Chemical context   

In recent years, great efforts have been devoted to the design and assembly of coordination polymers, not only because of the aesthetic beauty of their structures but also their potential applications in the fields of gas storage, separation, magnetism or their optical properties (Thangavelu *et al.*, 2015 ▸; Zhao *et al.*, 2014 ▸; Erer *et al.*, 2015 ▸; Eddaoudi *et al.*, 2015 ▸; O’Keeffe, 2009 ▸). The structural chemistry of transition metal halides with neutral N-donor co-ligands has been investigated thoroughly, leading to a multitude of complexes with new topologies and functionalities. Such N-donor ligands include, for example, tethering ligands such as bis­(4-pyridyl­meth­yl)piperazine (Low & LaDuca, 2015 ▸), 4,4′-di­pyridyl­amine (Brown *et al.*, 2008 ▸) or 4,4′-bi­pyridine (Lyons *et al.*, 2008 ▸). We are also inter­ested in conjugated terminal *N*-heterocyclic mol­ecules as ligands, which can endow the resulting structures with photoluminescent properties. 2,2′-Bi-1*H*-imidazole is used as such an important terminal N-donor co-ligand, which can not only direct the structural properties with hydrogen-bonding networks, but also can be used as a suitable fragment for π–π inter­actions through the imidazole rings.

We have explored the self-assembly of CdCl_2_ and 2,2′-bi-1*H*-imidazole in the presence of 2,2-di­methyl­succinic acid and obtained a new polymeric cadmium complex, [Cd(2,2′-bi-1*H*-imidazole)Cl_2_]_*n*_. Its crystal structure and luminescence sensing of solvent mol­ecules are reported in this communication.

## Structural commentary   

**Figure 1:**
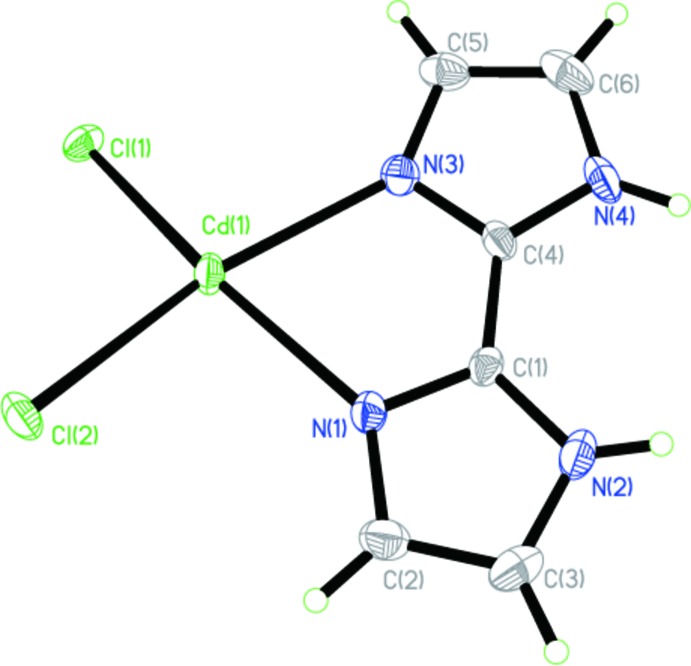
The asymmetric unit of the title compound, with anisotropic displacement parameters drawn at the 30% probability level.

**Figure 2:**
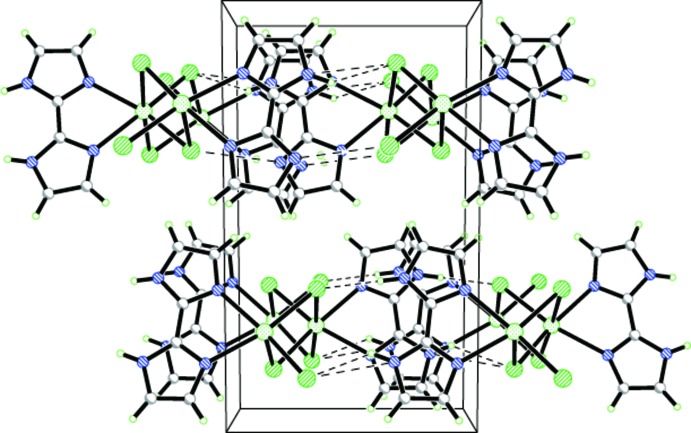
The supra­molecular structure showing the inter­actions between neighbouring chains. N—H⋯Cl hydrogen bonds are shown as dashed lines.

The asymmetric unit of the title compound is shown in Fig. 1 ▸. The central Cd^II^ atom is coordinated by four chloride ligands and two nitro­gen atoms from a chelating 2,2′-bi-1*H*-imidazole ligand, forming a distorted Cl_4_N_2_ octa­hedral coordination set (Fig. 2 ▸). The Cd—Cl and Cd—N bond lengths range from 2.5271 (11)–2.8150 (14) and 2.323 (3)–2.342 (4) Å, respectively. The five-membered Cd1/N1/C1/C2/N2 chelate ring is characterized by a bite angle of 72.6 (1)°. The two imidazole rings of the 2,2′-bi-1*H*-imidazole ligand are nearly parallel to each other, making a dihedral angle of 0.8 (5)°. The μ_2_-bridg­ing character of the four Cl ligands leads to the formation of a chain expanding parallel to the *c* axis (Fig. 2 ▸).
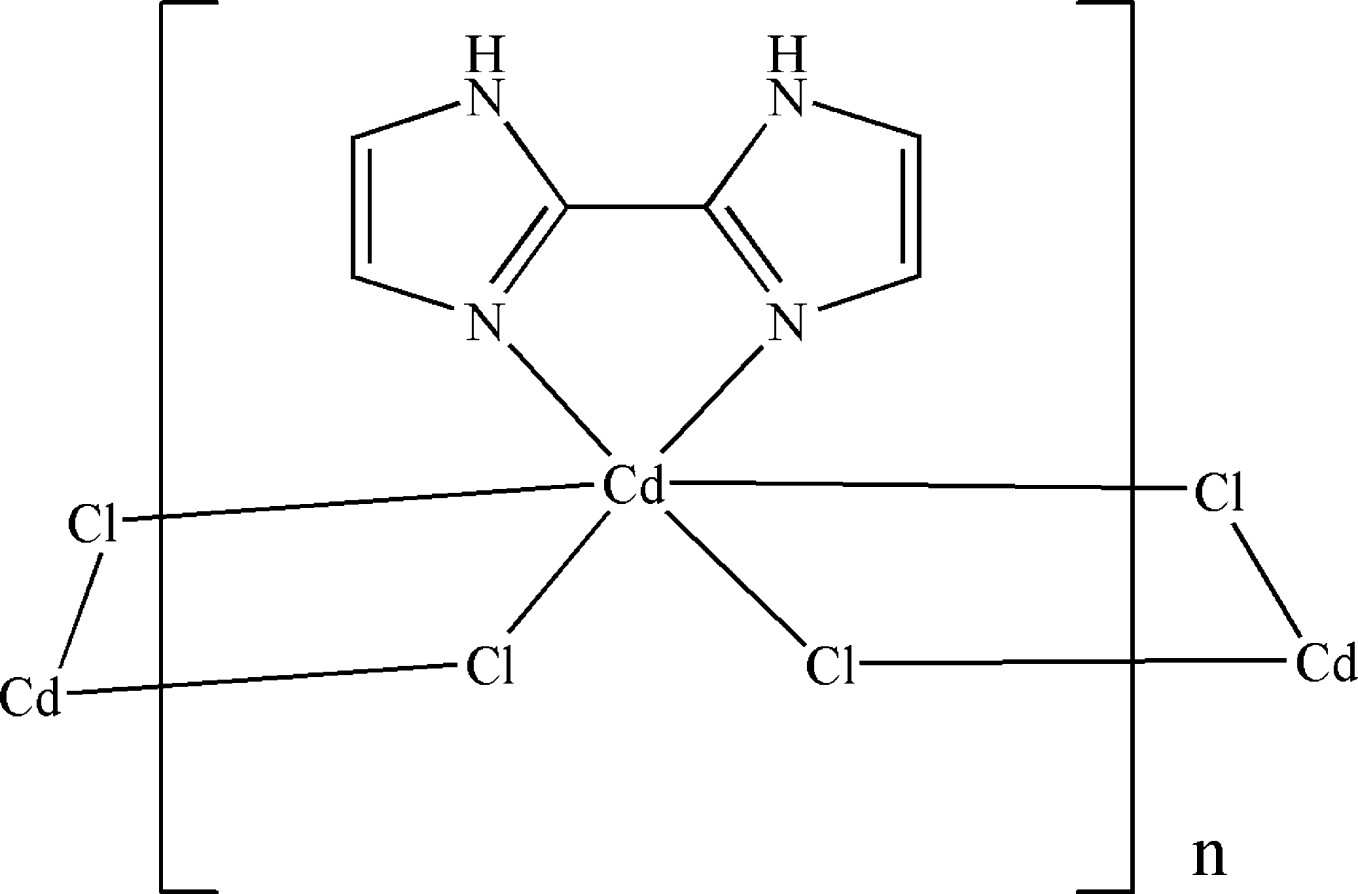


## Supra­molecular features   

In the presence of the chelating 2,2′-bi-1*H*-imidazole ligands that decorate the chains on both sides, the chains are directed by weak π–π inter­actions into zipper-like double-stranded chains with centroid-to-centroid distances of 3.6538 (15) and 3.9452 (14) Å, respectively. In addition, there are inter­molecular hydrogen bonds between the imidazole N atoms and coordinating Cl atoms of neighboring chains (Table 1 ▸). The π–π stacking inter­actions together with N—H⋯Cl hydrogen-bonding inter­actions expand the [CdCl_4/2_]_*n*_ chains to supra­molecular sheets parallel to the *bc* plane (Fig. 2 ▸).

## Luminescence properties   

**Figure 3:**
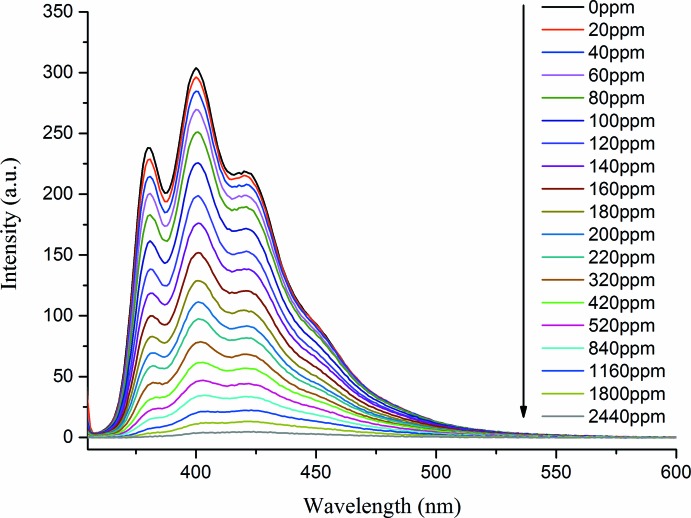
Fluorescence intensity of the title complex at different nitro­benzene concentrations in DMF.

Coordination polymers based on *d* ^10^ metal ions and conjugated organic ligands are promising candidates for potential photoactive materials with applications in chemical sensoring or in photochemistry. In particular, solvent-dependent quenching behaviour is of inter­est for the development of luminescent probes for chemical species (Liu *et al.*, 2015 ▸). Hence the luminescence properties of the title compound in different solvent emulsions were investigated. The luminescent intensities had no distinct differences if di­chloro­methane, aceto­nitrile, ethanol, ethyl acetate or benzene were selected as dispersing agents. However, the intensity had an abrupt decrease when the powdered samples of the title compound were dispersed in nitro­benzene. When the nitro­benzene solvent was gradually and increasingly added to the standard emulsions, the fluorescence intensities of the standard emulsions gradually decreased with increasing addition of nitro­benzene (Fig. 3 ▸). The fluorescence decrease was nearly proportional to the nitro­benzene concentration and intensity ultimately was found to be negligible. The efficient quenching of nitro­benzene in this system can be ascribed to the physical inter­action of the solute and solvent, which induces the electron transfer from the excited title compound to the electron-deficient nitro­benzene (Hao *et al.*, 2013 ▸). These results have given us the impetus to carry out more detailed investigations on the sensing behaviour of the title compound.

## Database survey   

A search of the Cambridge Structure Database (Version 5.35; last update May 2015; Groom *et al.*, 2016 ▸) for related Cd-based complexes with 2,2′-bi-1*H*-imidazole gave 41 hits. In most cases, 2,2′-bi-1*H*-imidazole serves as an ancillary ligand to be incorporated in carboxyl­ate coordination polymer systems. [Cd(2,2′-bi-1*H*-imidazole)Br_2_]_*n*_ has a very similar composition to the title compound and also shows an arrangement of polymeric chains constructed from the bridging behaviour of the Br ligand (Hester *et al.*, 1996 ▸); however, the space group is different (*C*2/*c*).

## Synthesis and crystallization   

A mixture of CdCl_2_·2.5H_2_O (0.5 mmol, 0.114 g), 2,2-di­methyl­succinic acid (0.5 mmol, 0.073 g), 2,2′-bi-1*H*-imidazole (0.5 mmol, 0.067 g) in water (8 ml) was stirred vigorously for 1 h at 333 K. Slow evaporation of the clear solution resulted in the separation of block-like colorless crystals as a pure phase. The crystals were washed with ethanol, and dried at room temperature. Calculated: C, 22.70; H, 1.90; N, 17.65; found: C, 22.51; H, 2.58; N, 17.49%.

## Refinement   

Crystal data, data collection and structure refinement details are summarized in Table 2 ▸. C-bound H atoms were positioned geometrically and constrained using a riding-model approximation, with C—H = 0.93 Å and *U*_iso_(H) = 1.2*U*_eq_(C). H atoms attached to the N atoms were found from difference maps but constrained with N—H = 0.86 Å and *U*_iso_(H) = 1.2*U*_eq_(N).

## Supplementary Material

Crystal structure: contains datablock(s) I. DOI: 10.1107/S2056989016013736/wm5319sup1.cif

Structure factors: contains datablock(s) I. DOI: 10.1107/S2056989016013736/wm5319Isup2.hkl

CCDC reference: 1501229

Additional supporting information: crystallographic information; 3D view; checkCIF report

## Figures and Tables

**Table 1:** Hydrogen-bond geometry (Å, °)

*D*—H⋯*A*	*D*—H	H⋯*A*	*D*⋯*A*	*D*—H⋯*A*
N2—H7⋯Cl2^i^	0.86	2.32	3.174 (4)	172
N4—H8⋯Cl1^i^	0.86	2.63	3.237 (4)	129

Symmetry code: (i) 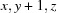.

**Table 2:** Experimental details

Crystal data	
Chemical formula	[CdCl_2_(C_6_H_6_N_4_)]
*M*_r_	317.45
Crystal system, space group	Monoclinic, *P*2_1_/*c*
Temperature (K)	296
*a*, *b*, *c* (Å)	14.977 (5), 8.777 (3), 7.160 (3)
β (°)	97.900 (5)
*V* (Å^3^)	932.3 (6)
*Z*	4
Radiation type	Mo *K*α
μ (mm^−1^)	2.87
Crystal size (mm)	0.26 × 0.21 × 0.17

Data collection	
Diffractometer	Bruker APEXII CCD area-detector
Absorption correction	Multi-scan (*SADABS*; Bruker, 2012 ▸)
*T*_min_, *T*_max_	0.523, 0.641
No. of measured, independent and observed [*I* > 2σ(*I*)] reflections	5643, 2229, 1997
*R*_int_	0.042
(sin θ/λ)_max_ (Å^−1^)	0.667

Refinement	
*R*[*F* ^2^ > 2σ(*F* ^2^)], *wR*(*F* ^2^), *S*	0.043, 0.113, 1.10
No. of reflections	2229
No. of parameters	119
H-atom treatment	H-atom parameters constrained
Δρ_max_, Δρ_min_ (e Å^−3^)	1.50, −1.62

Computer programs: *APEX2* and *SAINT* (Bruker, 2012 ▸), *SHELXS97*, *SHELXL97* and *SHELXTL* (Sheldrick, 2008 ▸).

## supplementary crystallographic information

### Crystal data

**Table:**

[CdCl_2_(C_6_H_6_N_4_)]	*F*(000) = 608
*M**_r_* = 317.45	*D*_x_ = 2.262 Mg m^−^^3^
Monoclinic, *P*2_1_/*c*	Mo *K*α radiation, λ = 0.71073 Å
Hall symbol: -P 2ybc	Cell parameters from 3238 reflections
*a* = 14.977 (5) Å	θ = 2.7–28.3°
*b* = 8.777 (3) Å	µ = 2.87 mm^−^^1^
*c* = 7.160 (3) Å	*T* = 296 K
β = 97.900 (5)°	Block, colorless
*V* = 932.3 (6) Å^3^	0.26 × 0.21 × 0.17 mm
*Z* = 4	

### Data collection

**Table:**

Bruker APEXII CCD area-detector diffractometer	2229 independent reflections
Radiation source: fine-focus sealed tube	1997 reflections with *I* > 2σ(*I*)
Graphite monochromator	*R*_int_ = 0.042
phi and ω scans	θ_max_ = 28.3°, θ_min_ = 2.7°
Absorption correction: multi-scan (*SADABS*; Bruker, 2012)	*h* = −12→19
*T*_min_ = 0.523, *T*_max_ = 0.641	*k* = −11→11
5643 measured reflections	*l* = −9→7

### Refinement

**Table:**

Refinement on *F*^2^	Secondary atom site location: difference Fourier map
Least-squares matrix: full	Hydrogen site location: inferred from neighbouring sites
*R*[*F*^2^ > 2σ(*F*^2^)] = 0.043	H-atom parameters constrained
*wR*(*F*^2^) = 0.113	*w* = 1/[σ^2^(*F*_o_^2^) + (0.0676*P*)^2^ + 0.4551*P*] where *P* = (*F*_o_^2^ + 2*F*_c_^2^)/3
*S* = 1.10	(Δ/σ)_max_ < 0.001
2229 reflections	Δρ_max_ = 1.50 e Å^−^^3^
119 parameters	Δρ_min_ = −1.62 e Å^−^^3^
0 restraints	Extinction correction: SHELXL97 (Sheldrick, 2008), Fc^*^=kFc[1+0.001xFc^2^λ^3^/sin(2θ)]^-1/4^
Primary atom site location: structure-invariant direct methods	Extinction coefficient: 0.044 (3)

### Special details

**Table:**

Geometry. All esds (except the esd in the dihedral angle between two l.s. planes) are estimated using the full covariance matrix. The cell esds are taken into account individually in the estimation of esds in distances, angles and torsion angles; correlations between esds in cell parameters are only used when they are defined by crystal symmetry. An approximate (isotropic) treatment of cell esds is used for estimating esds involving l.s. planes.
Refinement. Refinement of F^2^ against ALL reflections. The weighted R-factor wR and goodness of fit S are based on F^2^, conventional R-factors R are based on F, with F set to zero for negative F^2^. The threshold expression of F^2^ > 2sigma(F^2^) is used only for calculating R-factors(gt) etc. and is not relevant to the choice of reflections for refinement. R-factors based on F^2^ are statistically about twice as large as those based on F, and R- factors based on ALL data will be even larger.

### Fractional atomic coordinates and isotropic or equivalent isotropic displacement parameters (Å^2^)

**Table:**

	*x*	*y*	*z*	*U*_iso_*/*U*_eq_	
Cd1	0.23613 (2)	−0.15121 (3)	0.09150 (4)	0.02900 (17)	
Cl1	0.13506 (7)	−0.32441 (12)	−0.12634 (15)	0.0334 (2)	
Cl2	0.33418 (8)	−0.35735 (11)	0.28324 (18)	0.0385 (3)	
N1	0.3334 (2)	0.0418 (4)	0.2122 (5)	0.0313 (7)	
N2	0.3617 (3)	0.2854 (4)	0.2529 (6)	0.0381 (8)	
H7	0.3545	0.3826	0.2492	0.046*	
N3	0.1636 (2)	0.0810 (4)	0.0127 (5)	0.0317 (7)	
N4	0.1669 (3)	0.3302 (4)	0.0271 (6)	0.0403 (9)	
H8	0.1863	0.4213	0.0515	0.048*	
C1	0.3014 (3)	0.1809 (4)	0.1799 (6)	0.0271 (8)	
C2	0.4183 (3)	0.0601 (6)	0.3068 (7)	0.0415 (10)	
H2	0.4577	−0.0189	0.3468	0.050*	
C3	0.4364 (3)	0.2096 (6)	0.3338 (7)	0.0455 (11)	
H3	0.4893	0.2522	0.3952	0.055*	
C4	0.2120 (3)	0.2008 (5)	0.0758 (6)	0.0297 (8)	
C5	0.0842 (3)	0.1383 (6)	−0.0763 (7)	0.0410 (11)	
H5	0.0365	0.0801	−0.1346	0.049*	
C6	0.0854 (3)	0.2916 (7)	−0.0671 (7)	0.0489 (13)	
H6	0.0395	0.3577	−0.1157	0.059*	

### Atomic displacement parameters (Å^2^)

**Table:**

	*U*^11^	*U*^22^	*U*^33^	*U*^12^	*U*^13^	*U*^23^
Cd1	0.0339 (2)	0.0185 (2)	0.0330 (2)	−0.00127 (9)	−0.00124 (13)	−0.00016 (9)
Cl1	0.0291 (5)	0.0343 (5)	0.0368 (5)	−0.0095 (4)	0.0048 (4)	−0.0071 (4)
Cl2	0.0386 (6)	0.0319 (5)	0.0468 (6)	0.0149 (4)	0.0129 (5)	0.0112 (4)
N1	0.0334 (17)	0.0234 (15)	0.0358 (18)	−0.0003 (13)	0.0009 (13)	−0.0044 (13)
N2	0.044 (2)	0.0264 (18)	0.047 (2)	−0.0092 (15)	0.0150 (16)	−0.0079 (16)
N3	0.0334 (17)	0.0297 (17)	0.0320 (17)	0.0002 (14)	0.0049 (13)	0.0043 (14)
N4	0.048 (2)	0.0257 (17)	0.052 (2)	0.0143 (15)	0.0236 (19)	0.0099 (16)
C1	0.0300 (18)	0.0227 (16)	0.031 (2)	−0.0048 (15)	0.0129 (15)	−0.0025 (15)
C2	0.031 (2)	0.047 (3)	0.044 (2)	0.0051 (19)	−0.0024 (17)	−0.008 (2)
C3	0.037 (2)	0.053 (3)	0.046 (3)	−0.015 (2)	0.0044 (18)	−0.010 (2)
C4	0.0311 (19)	0.0236 (19)	0.037 (2)	0.0067 (16)	0.0151 (15)	0.0047 (16)
C5	0.030 (2)	0.054 (3)	0.038 (2)	0.0056 (18)	0.0020 (17)	0.0118 (19)
C6	0.043 (3)	0.057 (3)	0.048 (3)	0.023 (2)	0.013 (2)	0.019 (2)

### Geometric parameters (Å, º)

**Table:**

Cd1—N1	2.323 (3)	N3—C5	1.365 (5)
Cd1—N3	2.342 (4)	N4—C4	1.343 (5)
Cd1—Cl1	2.5271 (11)	N4—C6	1.354 (7)
Cd1—Cl2	2.6001 (12)	N4—H8	0.8600
Cd1—Cl1^i^	2.6944 (13)	C1—C4	1.450 (6)
Cd1—Cl2^ii^	2.8150 (14)	C2—C3	1.348 (8)
N1—C1	1.320 (5)	C2—H2	0.9300
N1—C2	1.365 (5)	C3—H3	0.9300
N2—C1	1.342 (5)	C5—C6	1.348 (7)
N2—C3	1.360 (7)	C5—H5	0.9300
N2—H7	0.8600	C6—H6	0.9300
N3—C4	1.321 (6)		
			
N1—Cd1—N3	72.61 (11)	C4—N3—Cd1	113.3 (3)
N1—Cd1—Cl1	163.82 (9)	C5—N3—Cd1	141.1 (3)
N3—Cd1—Cl1	98.98 (9)	C4—N4—C6	107.8 (4)
N1—Cd1—Cl2	91.80 (9)	C4—N4—H8	126.1
N3—Cd1—Cl2	160.47 (9)	C6—N4—H8	126.1
Cl1—Cd1—Cl2	98.87 (5)	N1—C1—N2	110.7 (4)
N1—Cd1—Cl1^i^	99.64 (9)	N1—C1—C4	119.3 (3)
N3—Cd1—Cl1^i^	87.70 (8)	N2—C1—C4	130.0 (4)
Cl1—Cd1—Cl1^i^	93.69 (4)	C3—C2—N1	109.9 (4)
Cl2—Cd1—Cl1^i^	83.31 (4)	C3—C2—H2	125.0
N1—Cd1—Cl2^ii^	84.49 (9)	N1—C2—H2	125.0
N3—Cd1—Cl2^ii^	93.59 (8)	C2—C3—N2	106.1 (4)
Cl1—Cd1—Cl2^ii^	82.24 (4)	C2—C3—H3	127.0
Cl2—Cd1—Cl2^ii^	96.60 (4)	N2—C3—H3	127.0
Cl1^i^—Cd1—Cl2^ii^	175.87 (3)	N3—C4—N4	110.5 (4)
Cd1—Cl1—Cd1^ii^	99.20 (4)	N3—C4—C1	120.3 (3)
Cd1—Cl2—Cd1^i^	94.46 (4)	N4—C4—C1	129.1 (4)
C1—N1—C2	105.6 (4)	C6—C5—N3	109.9 (5)
C1—N1—Cd1	114.5 (3)	C6—C5—H5	125.1
C2—N1—Cd1	139.8 (3)	N3—C5—H5	125.1
C1—N2—C3	107.6 (4)	C5—C6—N4	106.2 (4)
C1—N2—H7	126.2	C5—C6—H6	126.9
C3—N2—H7	126.2	N4—C6—H6	126.9
C4—N3—C5	105.6 (4)		
			
N1—Cd1—Cl1—Cd1^ii^	41.8 (3)	Cl2—Cd1—N3—C5	−140.3 (4)
N3—Cd1—Cl1—Cd1^ii^	99.06 (9)	Cl1^i^—Cd1—N3—C5	−77.8 (4)
Cl2—Cd1—Cl1—Cd1^ii^	−88.90 (4)	Cl2^ii^—Cd1—N3—C5	98.2 (4)
Cl1^i^—Cd1—Cl1—Cd1^ii^	−172.70 (4)	C2—N1—C1—N2	−1.1 (5)
Cl2^ii^—Cd1—Cl1—Cd1^ii^	6.62 (3)	Cd1—N1—C1—N2	−179.6 (2)
N1—Cd1—Cl2—Cd1^i^	93.14 (9)	C2—N1—C1—C4	178.4 (4)
N3—Cd1—Cl2—Cd1^i^	56.8 (3)	Cd1—N1—C1—C4	−0.2 (4)
Cl1—Cd1—Cl2—Cd1^i^	−99.06 (4)	C3—N2—C1—N1	0.8 (5)
Cl1^i^—Cd1—Cl2—Cd1^i^	−6.35 (3)	C3—N2—C1—C4	−178.6 (4)
Cl2^ii^—Cd1—Cl2—Cd1^i^	177.80 (3)	C1—N1—C2—C3	1.0 (5)
N3—Cd1—N1—C1	0.5 (3)	Cd1—N1—C2—C3	178.9 (3)
Cl1—Cd1—N1—C1	61.0 (5)	N1—C2—C3—N2	−0.5 (5)
Cl2—Cd1—N1—C1	−167.6 (3)	C1—N2—C3—C2	−0.1 (5)
Cl1^i^—Cd1—N1—C1	−84.0 (3)	C5—N3—C4—N4	−1.1 (4)
Cl2^ii^—Cd1—N1—C1	96.0 (3)	Cd1—N3—C4—N4	−179.7 (3)
N3—Cd1—N1—C2	−177.4 (5)	C5—N3—C4—C1	179.6 (4)
Cl1—Cd1—N1—C2	−116.8 (4)	Cd1—N3—C4—C1	0.9 (4)
Cl2—Cd1—N1—C2	14.6 (4)	C6—N4—C4—N3	1.5 (5)
Cl1^i^—Cd1—N1—C2	98.1 (4)	C6—N4—C4—C1	−179.2 (4)
Cl2^ii^—Cd1—N1—C2	−81.9 (4)	N1—C1—C4—N3	−0.5 (6)
N1—Cd1—N3—C4	−0.7 (2)	N2—C1—C4—N3	178.8 (4)
Cl1—Cd1—N3—C4	−166.5 (2)	N1—C1—C4—N4	−179.8 (4)
Cl2—Cd1—N3—C4	37.6 (4)	N2—C1—C4—N4	−0.5 (7)
Cl1^i^—Cd1—N3—C4	100.1 (3)	C4—N3—C5—C6	0.3 (5)
Cl2^ii^—Cd1—N3—C4	−83.8 (3)	Cd1—N3—C5—C6	178.3 (3)
N1—Cd1—N3—C5	−178.7 (5)	N3—C5—C6—N4	0.6 (6)
Cl1—Cd1—N3—C5	15.5 (5)	C4—N4—C6—C5	−1.2 (5)

Symmetry codes: (i) *x*, −*y*−1/2, *z*+1/2; (ii) *x*, −*y*−1/2, *z*−1/2.

### Hydrogen-bond geometry (Å, º)

**Table:**

*D*—H···*A*	*D*—H	H···*A*	*D*···*A*	*D*—H···*A*
N2—H7···Cl2^iii^	0.86	2.32	3.174 (4)	172
N4—H8···Cl1^iii^	0.86	2.63	3.237 (4)	129

Symmetry code: (iii) *x*, *y*+1, *z*.
